# Supplementary material for: Neuronal Cannabinoid CB1 Receptors Suppress the Growth of Melanoma Brain Metastases by Inhibiting Glutamatergic Signalling
Source: Cancers (Basel). 2023 Apr 24;15(9):2439. doi: 10.3390/cancers15092439 (PMC10177062; doi:10.3390/cancers15092439)

Figure 5B, Glu-CB$_1$R (WT and KO)

pNMDAR2B-Y1252 (Mr ~170 KDa)

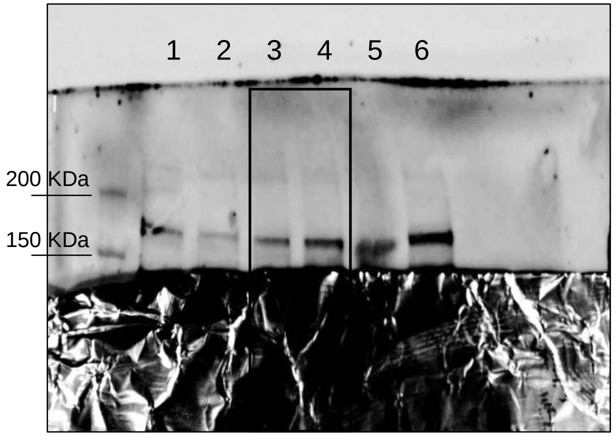

An aluminium foil was placed to allow a longer exposure of the specific band (see right)

Resizing

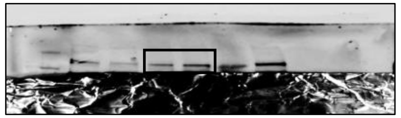

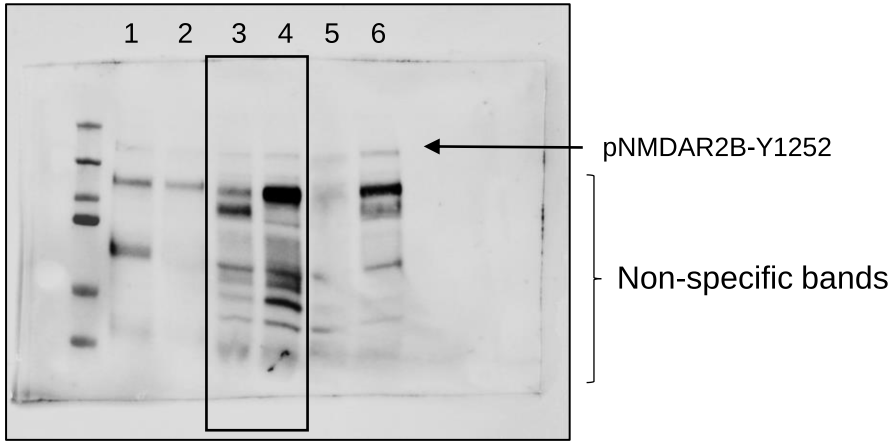

Shorter exposure without aluminium foil

Key to blot lanes

1. Unrelated sample
2. Unrelated sample
3. Glu-CB$_1$R WT tumour
4. Glu-CB$_1$R KO tumour
5. Glu-CB$_1$R WT tumour
6. Glu-CB$_1$R KO tumour

Tubulin (Mr ~55 KDa)

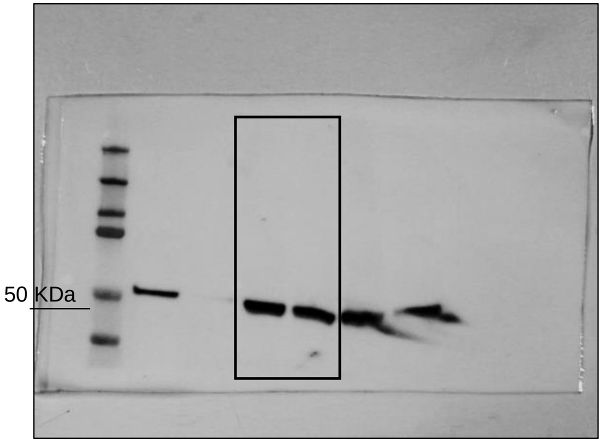

Resizing

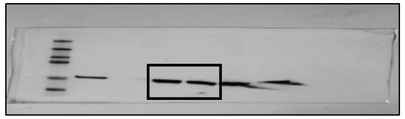

Figure 5B, GABA-CB$_1$R (WT and KO)

pNMDAR2B-Y1252 (Mr ~170 KDa)

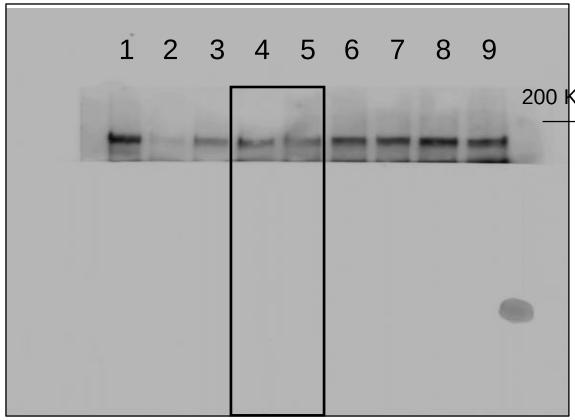

Resizing

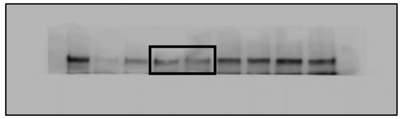

The membrane was cut to allow the simultaneous detection of tubulin

Key to blot lanes

1. Unrelated sample
2. Unrelated sample
3. Unrelated sample
4. GABA-CB$_1$R WT tumour
5. GABA-CB$_1$R KO tumour
6. GABA-CB$_1$R WT tumour
7. GABA-CB$_1$R KO tumour
8. GABA-CB$_1$R WT tumour
9. GABA-CB$_1$R KO tumour

Tubulin (Mr ~55 KDa)

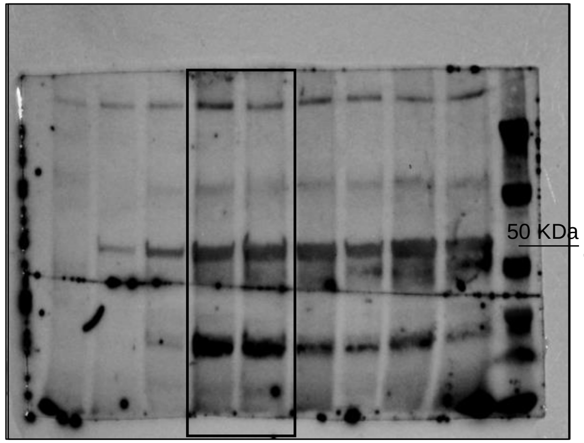

Resizing

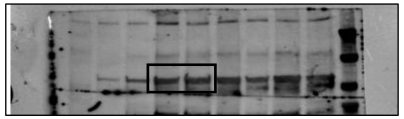

Supplement: Supplementary file 1 [file cancers-15-02439-s001.zip › Figure S1-Uncropped scans of all blots of the study.pdf]
